# Whole genome sequencing and characteristics of *Escherichia coli* with co-existence of ESBL and *mcr* genes from pigs

**DOI:** 10.1371/journal.pone.0260011

**Published:** 2021-11-16

**Authors:** Suthathip Trongjit, Rungtip Chuanchuen

**Affiliations:** Research Unit in Microbial Food Safety and Antimicrobial Resistance, Department of Veterinary Public Health, Faculty of Veterinary Science, Chulalongkorn University, Bangkok, Thailand; Zhejiang University, CHINA

## Abstract

This study aimed to analyze three ESBL-producing *E*. *coli* co-harboring *mcr* and ESBL genes from a healthy fattening pig (E. 431) and two sick pigs (ECP.81 and ECP.82) in Thailand using Whole Genome Sequencing (WGS) using either Illumina MiSeq or HiSeq PE150 platforms to determine their genome and transmissible plasmids. E. 431 carrying *mcr-2*.*1* and *mcr-3*.*1* belonged to serotype O142:H31 with ST29 sequence type. ECP.81 and ECP.82 from sick pigs harboring *mcr-1*.*1* and *mcr-3*.*1* were serotype O9:H9 with ST10. Two *mcr-1*.*1* gene cassettes from ECP.81 and ECP.82 were located on IncI2 plasmid with 98% identity to plasmid pHNSHP45. The *mcr-2*.*1*-carrying contig in E. 431 showed 100% identity to plasmid pKP37-BE with the upstream flanking sequence of IS*1595*. All three *mcr-3*.*1*-carrying contigs contained the ΔTn*As2*-*mcr-3*.*1*-*dgkA* core segment and had high nucleotide similarity (85–100%) to *mcr-3*.*1*-carrying plasmid, pWJ1. The mobile elements i.e. IS*4321*, ΔTn*As2*, IS*Kpn40* and IS*3* were identified in the flanking regions of *mcr-3*. Several genes conferring resistance to aminoglycosides (*aac(3)-IIa*, *aadA1*, *aadA2b*, *aph(3’’)-Ib*, *aph(3’)-IIa* and *aph(6)-Id*), macrolides (*mdf*(*A*)), phenicols (*cmlA1*), sulphonamide (*sul3*) and tetracycline (*tet(A) and tet(M)*) were located on plasmids, of which their presence was well corresponded to the host’s resistance phenotype. Amino acid substitutions S83L and D87G in GyrA and S80I and E62K in ParC were observed. The *bla*_CTX-M-14_ and *bla*_CTX-M-55_ genes were identified among these isolates additionally harbored *bla*_TEM-1B_. Co-transfer of *mcr-1*.*1*/*bla*_TEM-1B_ and *mcr-3*.*1*/*bla*_CTX-M-55_ was observed in ECP.81 and ECP.82 but not located on the same plasmid. The results highlighted that application of advanced innovation technology of WGS in AMR monitoring and surveillance provide comprehensive information of AMR genotype that could yield invaluable benefits to development of control and prevention strategic actions plan for AMR.

## Introduction

Antimicrobial resistance (AMR) in bacteria is one the serious public health threats worldwide and has been complicated by emerging and spread of bacterial pathogens that are resistant to multiple drugs as well as clinically important antimicrobials (CIA) for human medicine. In the past decades, resistance to last-resort antibiotics such as carbapenems and polymyxins has been increasingly reported in Gram-negative bacteria, especially, emergence of carbapenem resistant Enterobacteriaceae (CRE) [1]. This has raised a particular concern of limited treatment antibiotic for bacterial infection and the need for discovering novel powerful antibiotics for future treatment [2].

Colistin is a last-resort antibiotic, especially for the treatment of infections with CRE in humans [3] and classified into the Highest Priority Critically Important Antimicrobials (HPCIA) for human medicine by WHO [4]. However, it has been widely used in livestock production, especially in pigs, for a long time. The global survey of colistin usage varied in different countries [5]. For example, the US government has prohibited colistin use in animal production and human medicine due to its nephrotoxicity and neurotoxicity [6]. China is considered to be the world’s highest user of colistin in agriculture [5], however presently colistin has been banded for using as a feed additive for animals since 2016 [7]. In EU, the use of colistin in Germany, Portugal, Italy and Estonia was more extensive in than other European countries [5]. In Thailand, approximately 40 tons of colistin was used per year as medicated feed mills for prevention and/or treatment of post-weaning diarrhea (PWD) [8].

Resistance to colistin is attributed to chromosomal mutations in the PhoPQ two-component regulatory system and *pmr*CAB operon, resulting in the modification of the lipid A of lipopolysaccharides [9]. In 2015, plasmid-borne gene, *mcr-1*, encoding a phosphoethanolamine transferase was found to be common in Enterobacteriaceae isolated from animals, food stuffs and patients in China [10]. The report has attracted global concern of its high efficiency of horizontal transfer that places *mcr-1* as a potential public health threat. Up to date, nine different *mcr* variants (*mcr-1* to *mcr-10)* have been reported and were isolated from bacteria of different origins, including humans, livestock, wildlife, and environmental samples [10–14]. A previous study indicated that the spread of *mcr* genes mainly associated with epidemic plasmid replicon types of various size (e.g. IncI2, IncHI1, IncHI2, IncP, IncFIB and IncX4) [15].

*Escherichia coli* plays an important role in spread of AMR due to its capacity to accumulate AMR genes that can be horizontally transmitted to other bacterial species. Previous studies indicated that the *E*. *coli* isolates, particularly the sequence types ST10 and ST155, from food animal served as an important reservoir of *mcr-1* [11, 16]. Previously, *E*. *coli* with coexistence of *mcr-1* and *bla*_VIM-1_ [17] or *bla*_CTX-M-55_ [18] on a single plasmid was isolated. This could be a serious public health threat potentially leading to pan-drug resistant infection. Moreover, the IS*Apl1* insertion sequence has been considered a key element mediating translocation of *mcr-1* into diverse types of plasmid and was localized on the chromosome by forming circular intermediates [19]. A previous study confirmed that the presence of mobile elements, including IS*4321*, ΔTn*As2* or IS*Kpn40*, in the flanking regions of *mcr-3* in *E*. *coli* [20]. Genetic characterization of the plasmid backbones of *mcr* genes and other mobile genetic elements (i.e IS and Tn) is required with the expectation to improve the understanding of the molecular mechanisms underlying dissemination of *mcr* variants.

Next generation sequencing (NGS) appears to be a very useful tool for epidemiological surveillance and characterization of AMR [21]. Due to its ability to provide comprehensive genomic data in a single time, NGS has been increasingly used for genomic characterization of foodborne bacterial pathogens, identification of clonal groups in bacteria of public health importance and molecular characterization of epidemic plasmids harboring AMR or/and virulence genes [22]. We previously isolated *E*. *coli* with coexistence of ESBL and *mcr* genes from healthy and sick pigs [23]. In this study, three *E*. *coli* co-harboring ESBL and *mcr* genes were characterized by using WGS approach.

## Materials and methods

### Epidemiological background of *E*. *coli*

In this study, three *E*. *coli* isolates co-expressing ESBL and *mcr* genes (i.e. E.431, ECP.81 and ECP.82) were obtained from our bacterial collection (Trongjit et al, 2020) [23]. E.431 was collected in 2007 from a healthy fattening pig and carries *mcr-2*, *mcr-3* and *bla*_CTX-M14_. The other two *E*. *coli* isolates carrying *mcr-1* and *mcr-3* and simultaneously containing *bla*_CTX-M-14_/*bla*_CTX-M-55_/*bla*_TEM-1B_ (ECP.81) and *bla*_CTX-M-14_/*bla*_TEM-1B_ (ECP.82) were isolated from two different clinically sick pigs in 2013.

These isolates were collected as part of the molecular study of colistin resistant and ESBL-producing *E*. *coli* obtained from healthy (n = 354) and clinically sick pigs (n = 100) in Thailand [23]. Antimicrobial susceptibilities were determined using two-fold agar dilution method [24] and ESBL production was examined using disk diffusion method [24]. The results for all antimicrobials were interpreted using CLSI breakpoints [24], except EUCAST breakpoints was used for colistin [25]. All were PCR screened for colistin-resistance genes including *mcr-1* [10], *mcr-2* [26], *mcr-3* [11] and *mcr-4* [27] and ß-lactamase-encoding genes including *bla*_TEM_, *bla*_PSE-M_, *bla*_SHV,_ *bla*_CTX−M_, *bla*_CMY-1_ and *bla*_CMY-2_ [28–30]. All PCR primers used in this study are listed in S1 Table.

### Conjugation experiment

Biparental filter mating experiment was conducted to confirm the localization of *mcr* and ESBL genes on conjugative plasmid [31] in E. 431, ECP.81 and ECP.82. The plasmid-free SE12Rif^R^, spontaneous rifampicin-resistant *Salmonella* Enteritidis (rifampicin MIC = 256 μg/ml), was used as recipient [31]. Transconjugants were confirmed to be *Salmonella* on Xylose Lysine Deoxycholate agar (Difco, MD, USA) containing 32 μg/mL rifampicin and an appropriate antibiotic (i.e. 100 μg/mL ampicillin and/or 2 μg/mL colistin). The presence of *mcr* (n = 3) and ESBL (n = 3) genes in transconjugants was determined by PCR using specific primers as described above.

### DNA preparation and Whole Genome Sequencing (WGS)

Genomic DNA of E. 431, ECP.81 and ECP.82 were extracted by using the QIAamp DNA Mini Kit (Qiagen, Hilden, Germany). Plasmid DNA of ECP.81T and ECP.82T, the transconjugants obtained from ECP. 81 and ECP.82, were extracted by using Qiagen Plasmid Maxi Kit (Qiagen). The amount of all DNA samples was quantified according to Illumina sequencing sample requirements. The DNA samples were dissolved in 10mM Tris buffer to obtained at least 10 nM in 10 μl of minimum volume. The purity of DNA was determined at A260/280 and A260/230 using a Nanodrop ND-1000 spectrophotometer (Thermo Fisher Scientific, Delaware, USA).

The quantified DNA were subjected to Whole Genome Sequencing (WGS) by using Illumina platform MiSeq or HiSeq PE150 (Illumina, San Diego, CA, US). The libraries were prepared by using Nextera XT sample preparation kit and sequenced with 2×250 or 350 paired-end reads protocol on an Illumina platform (MiSeq or HiSeq PE150) at Omics Sciences and Bioinformatics Center (OSBC), Faculty of Science, Chulalongkorn University and Singapore Joint Venture & Sequencing Center Novogen AIT.

### Analysis of DNA sequence data

Trimming of raw sequence reads was performed using the CLC Genomics Workbench software version 11.0.0 (CLC bio, Aarhus, Denmark) with default settings. De novo assembly of the trimmed reads was conducted using CLC Genomics Workbench or SPAdes 3.5.0 software [32]. Identification of Open Reading Frames (ORFs) and genome annotation of the assembled genetic elements was performed by using Prokka [33] and/or PATRIC 3.6.5 [34] with default settings.

WGS data sets were analyzed using Open-access bioinformatic webtool available at the Center for Genomic Epidemiology (http://www.genomicepidemiology.org/). *In silico* typing based on WGS of assembled genomes/contigs in FASTA format was carried out by using Serotype Finder 2.0 [35] with selected threshold of 90% identity and 60% total serotype gene length. Multi-Locus Sequence Typing (MLST 2.0) was applied for molecular typing of *E*. *coli* [36]. The MLST allele sequences and profile data used in MLST 2.0 were obtained from https://www.PubMLST.org and determined at 90% identity and 60% minimum length. The core genome MLST (cgMLST) profile of these isolates were investigated using cgMLSTFinder 1.1 from CGE databases [37]. Res Finder 4.1 [38] and Point Finder [39] were used to predict AMR genes and chromosomal point mutations from genomic sequences based on 90% identity. Plasmid replicon sequence analysis and identification of virulence genes were performed using Plasmid Finder 2.1 [40] and Virulence Finder 2.0 [41], respectively with the threshold of 90% identity and 60% minimum length. Genome assemblies of three *E*. *coli* isolates were further analyzed for genomic relatedness using the *E*. *coli* cgMLST scheme available at the BacWGSTdb 2.0 (http://bacdb.cn/BacWGSTdb/) [42].

### Plasmid and phylogenetic analysis

Plasmid reconstruction from WGS data was conducted by using highly homologous complete plasmid sequence references available in NCBI. The alignment and assembly of sequences was performed using CLC Genomics Workbench or PATRIC 3.6.5 [34]. Plasmid sequences were annotated by Prokka [33] or PATRIC 3.6.5 [34] and manually edited. Then, annotated plasmid sequences were analyzed. Circular comparison between IncI2 plasmid carrying *mcr-1* and most identical reference plasmids available at NCBI database was generated by CG view server [43]. All reference plasmids used for comparison in this study and obtained from NCBI database are listed in (S1 and S2 Tables).

The phylogenetic three of the *mcr-1*.*1*-carrying IncI2 plasmids was analyzed by comparing the two *mcr-1*.*1*-carrying IncI2 plasmids in ECP81 and ECP82 with 20 of *mcr-1*-carrying IncI2 plasmids from *E*. *coli* deposited in the GenBank database. The sequences were aligned using MUSCLE and phylogenetic interferences were obtained using the neighbor-joining method within the MEGA 10 software at set up of 1000 times bootstrap values to generate a majority consensus tree. The phylogenetic relationship of the core genome segment of *mcr-3* carrying contigs was conducted by MEGA 10.0 program with maximum likelihood method (1,000 bootstrap replicates). All *mcr-3* harboring contig sequences used to produce the phylogenetic trees were generated in this study and the *mcr-3* reference genome sequences were obtained from the GenBank database (S2 Table).

### Nucleotide sequence accession number

The complete sequences of *mcr-1* carrying IncI2 plasmids from ECP81 and ECP82 in this study have been deposited at in GenBank under the accession numbers OK323956 and OK323955, respectively. Partial sequences of *mcr-3*-bearing plasmids from E431, ECP81 and ECP82 and partial *mcr-2* sequence from E431 were deposited in GenBank database with the accession of OK323954, OK323952, OK323953 and OK323951, respectively.

## Results

### Antimicrobial susceptibilities and molecular characteristics

E.431 from healthy pig and ECP.81 and ECP.82 from sick pigs exhibited multidrug resistance (MDR) phenotype (resistance to at least three different antimicrobial classes). E. 431 from healthy pig was resistant to all antibiotics tested, except trimethoprim (Table 1). ECP. 81 was resistant to all antimicrobials tested while the other EC.P. 82 remained susceptible to trimethoprim and ceftazidime.

**Table 1:** Antimicrobial susceptibilities of ESBL-producing *E*. *coli* carrying *mcr* from pigs in Thailand (n = 3).

Antimicrobial agent	Healthy pig			Clinically ill pig		
	E. 431		ECP. 81		ECP. 82	
	MIC (μg/mL)	S/R	MIC (μg/mL)	S/R	MIC (μg/mL)	S/R
**Polymyxin**						
Colistin	8	R	8	R	8	R
**Beta-lactams**						
Ampicillin	>512	R	>512	R	>512	R
Cefotaxime	32	R	32	R	64	R
Cefotaxime/clavulanic acid	0.12	ESBL^a^	0.12	ESBL^a^	0.12	ESBL^a^
Ceftazidime	2	R	1	S	1	S
Ceftazidime/clavulanic acid	≤0.12	ESBL^a^	0.25	ESBL^a^	0.25	ESBL^a^
Cefepime	8	I	8	I	4	I
Cefoxitin	2	S	8	S	8	S
Ertapenem	≤0.015	S	0.03	S	0.03	S
Imipenem	≤0.12	S	≤0.12	S	≤0.12	S
Meropenem	≤0.03	S	≤0.03	S	≤0.03	S
**Aminoglycosides**						
Streptomycin	32	R	256	R	256	R
Gentamicin	1024	R	256	R	256	R
**Fluoroquinolones**						
Ciprofloxacin	128	R	32	R	32	R
**Phenicol**						
Chloramphenicol	64	R	256	R	128	R
**Tetracycline**						
Tetracycline	1	S	256	R	256	R
**Folate pathway inhibitors**						
Sulfamethoxazone	256	S	>2048	R	>2048	R
Trimetroprim	64	R	1024	R	1	S

^a^ = indicate ESBL production.

S, Susceptible.

I, Intermediate.

R, Resistant.

Genome size of E431 was 5,863,412 bp with GC content of 50.07%. The genome size of ECP81 and ECP82 was 5,402,991bp and 5,579,866 bp, respectively, with 50.2% GC content. Based on in silico typing and MLST, E. 431 belonged to serotype O142:H32 with sequence type ST29. The other two *E*. *coli* isolates, ECP.81 and ECP.82 were serotype O9:H9 belonging to ST10.

The cgMLST scheme based on the presence or absence of 2,513 genes in E431, ECP81 and ECP82 were assigned into cg13958, cg40289 and cg 136327, respectively. Based on the cgMLST scheme, ECP81 and ECP82 had high genomic similarity to *E*. *coli* strain TZ7_Sa from a pig in Australia (Accession no, NZ_SAIB01) with distance of 324 and 287 different alleles, respectively and *E*. *coli* strain STEC_545 isolated from a diarrhea patient in the Netherlands (Accession no, LODB01) with different alleles of 349 and 309, respectively. Both the TZ7_Sa and STEC_545 were ST10 *E*. *coli* strains and carried similar AMR genes detected in ECP81 and ECP82 (i.e. *aadA1*, *aadA2*, *bla*_TEM-1B_, *cmlA1*, *dfrA12*, *mdf(A)*, *sul3*, in TZ7_Sa and *mdf(A)* in STEC_545). However, neither *mcr* nor ESBL genes were detected in the TZ7_Sa. No closest-genomic similarity in the database was observed for E431.

Plasmid finder showed that all the isolates harbored four plasmid replicons, including IncFIB, IncFII, IncHI2 and Col440I. E. 431 additionally carried Col156 and P0111 replicon types, while ECP.81 and ECP.82 additionally contained ColpVC, IncI1-I(γ) and IncI2 replicon types (Table 2).

**Table 2:** Molecular characteristics of ESBL-producing *E*. *coli* carrying *mcr* from pigs in Thailand (n = 3).

Strain	Source	Serotype	MLST	Plasmid content	Virulence profile	Resistance genes			Amino acid change in in GyrA and/or ParC
						*mcr*	ß-lactamase	Others by WGS	
**E. 431**	Healthy pig	O142:H31	29	Col156, Col440II, IncFIB, IncFII, IncHI2, P0111	*ast*A, *celb*, *cif*, *eae*, *efa*1, *esp*A, *esp*B, *esp*F, *esp*J, *gad*, *hra*, *iss*, *iuc*C, *iut*A, *kat*P, *lpf*A, *nle*A, *nle*B, *nel*C, *omp*T, *sep*A, *ter*C, *tox*B, *tra*T, *tsh*	*mc*r *2*.*1*, *mcr 3*.*1*	*bla*_CTX-M-14_	*aac(3)-IIa*, *aadA1*, *aadA2b*, *aph(3’’)-Ib*, *aph(3’)-IIa*, *aph(6)-Id*, *mdf(A)*, *cmlA1*, *oqxA*, *oqxB*, *sul3*, *tet(A)*, *tet(M)*	GyrA (S83L, D87G), ParC (S80I)
**ECP. 81**	Sick pig	O9:H9	10	Col440I, ColpVC, IncFIB, IncFII, IncHI2, IncI1-I(γ), IncI2	*ast*A, *fed*A, *fed*F, *gad*, *hra*, *sta1*, *ter*C, *tra*T	*mc*r *1*.*1*, *mcr 3*.*1*	*bla*_CTX-M-14_, *bla*_CTX-M-55_, *bla*_TEM-1B_	*aac(3)-IIa*, *aadA1*, *aadA2b*, *aph(3’’)-Ib*, *aph(3’)-IIa*, *aph(6)-Id*, *cfr*, *lnu(F)*, *mdf(A)*, *catA2*, *cmlA1*, *oqxA*, *oqxB*, *qnrS13*, *sul2*, *sul3*, *tet(A)*, *tet(M)*	GyrA (S83L), ParC (S80I)
**ECP. 82**	Sick pig	O9:H9	10	Col440I, ColpVC, IncFIB, IncFII, IncHI2, IncI1-I(γ), IncI2	*ast*A, *fed*A, *fed*F, *gad*, *hra*, *ter*C	*mc*r *1*.*1*, *mcr 3*.*1*	*bla*_CTX-M-14_, *bla*_TEM-1B_	*aac(3)-IIa*, *aadA1*, *aadA2b*, *aph(3’’)-Ib*, *aph(3’)-IIa*, *aph(6)-Id*, *mdf(A)*, *cmlA1*, *oqxA*, *oqxB*, *qnrS13*, *sul2*, *sul3*, *tet(A)*, *tet(M)*	GyrA (S83L), ParC (S80I, E62K)

Identification of virulence profiles revealed that E. 431, ECP.81 and ECP.82 harbored similar virulence genes including *ast*A encoding heat stable toxin, *fed*A/*fed*F encoding fimbria adhesin, *gad* encoding glutamate decarboxylase, *hra* encoding heat resistance agglutinin, *ter*C encoding tellurium ion resistance protein and *tra*T encoding outer membrane protein resistance. The presence of certain virulence genes including *celb*, *cif*, *eae*, *efa*1, *esp*A, *iss*, *iuc*C, *iut*A, *kat*P, *lpf*A, *nle*A, *nle*B, *nel*C, *omp*T, *sep*A, *tox*B and *tsh* varied among the three isolates (Table 2).

### Presence and horizontal transfer of AMR determinants

WGS data analysis using Resfinder revealed the presence of AMR genes that were consistent with their resistance phenotype (Table 2). E.431 caried *mcr2*.*1*, *mcr3*.*1* and *bla*_CTX-M-14_. Both ECP.81 and ECP.82 carried *mcr1*.*1* and *mcr3*.*1* genes. ECP.81 additionally harbored *bla*_CTX-M-14_, *bla*_CTX-M-55_ and *bla*_TEM-1B_, while ECP.82 simultaneously carried *bla*_CTX-M-14_ and *bla*_TEM-1B_. In addition to ESBL and *mcr* genes, all three isolates concurrently contained various AMR genes conferring (but not limited) resistance to aminoglycoside (*aac(3)-IIa*, *aadA1*, *aadA2b*, *aph(3’’)-Ib*, *aph(3’)-IIa* and *aph(6)-Id*), macrolide (*mdf*(*A*)), phenicol (*cmlA1*), sulphonamide (*sul3*) and tetracycline (*tet(A) and tet(M)*) that were consistent with the AMR phenotypes. The single point mutations in quinolone resistant-determining regions (QRDR) of *gyrA* and *parC* resulting in amino acid substitution in GyrA and ParC were identified including S83L and D87G in GyrA and S80I and E62K in ParC. Plasmid mediated quinolone resistance genes (PMQR) i.e. *oqxA*, *oqxB and qnrS13* were detected in ECP.81 and ECP.82.

Based on conjugation experiment, only ECP.81 and ECP.82 were able to transfer *mcr* and ß-lactamase genes to SE12Rif^R^. Using ampicillin and rifampicin as selective pressure, ECP.81 conjugally transferred *bla*_CTX-M-55_ together with *mcr-3*.*1* and *aac(3)-IIa*. The NGS analysis of plasmid revealed the presence of Col440I, ColpVC, IncFIB and IncFII plasmid replicons in the transcojugant ECP.81T. ECP.82 was capable of transfer *bla*_TEM-1B_, *mcr 1*.*1*, *aac(3)-IIa*, *aadA2b*, *aph(3’’)-Ib*, *aph(6)-Id*, *qnrS13* and *sul2* at the same time. Several plasmid replicon types were identified in ECP.82T, including IncI2, Col440I, ColpVC, IncFIB, IncFII and IncI1(γ) (Table 3). Under colistin and rifampicin selection pressure, only ECP.82 could transfer *mcr-3* to the recipient strain.

**Table 3:** Antimicrobial resistance genes and plasmid contents in donors and transconjugants revealed by NGS.

Strain	Role	AMR gene	Plasmid replicon type
**EC.P. 81**	Donor	*mc*r *1*.*1*, *mcr 3*.*1*, *bla*_CTX-M-14_, *bla*_CTX-M-55_, *bla*_TEM-1B,_ *aac(3)-IIa*, *aadA1*, *aadA2b*, *aph(3’’)-Ib*, *aph(3’)-IIa*, *aph(6)-Id*, *cfr*, *lnu(F)*, *mdf(A)*, *catA2*, *cmlA1*, *oqxA*, *oqxB*, *qnrS13*, *sul2*, *sul3*, *tet(A)*, *tet(M)*	Col440I, ColpVC, IncFIB, IncFII, IncHI2, IncI1-I(γ), IncI2
**EC.P. 81T**	Transconjugant	*mcr-3*.*1*, *bla*_CTX-M-55_ *aac(3)-IIa*	Col440I, ColpVC, IncFIB, IncFII
**EC.P. 82**	Donor	*mc*r *1*.*1*, *mcr 3*.*1*, *bla*_CTX-M-14_, *bla*_TEM-1B,_ *aac(3)-IIa*, *aadA1*, *aadA2b*, *aph(3’’)-Ib*, *aph(3’)-IIa*, *aph(6)-Id*, *mdf(A)*, *cmlA1*, *oqxA*, *oqxB*, *qnrS13*, *sul2*, *sul3*, *tet(A)*, *tet(M)*	Col440I, ColpVC, IncFIB, IncFII, IncHI2, IncI1-I(γ), IncI2
**EC.P. 82T**	Transconjugant	*mc*r *1*.*1*, *bla*_TEM-1B_ *aac(3)-IIa*, *aadA2b*, *aph(3’’)-Ib*, *aph(6)-Id*, *qnrS13*, *sul2*	Col440I, ColpVC, IncFIB, IncFII, IncI1-I(γ), IncI2

### *mcr*-harboring plasmids

The *mcr 1*.*1* gene was identified on IncI2 plasmid in ECP.81 (contig 30; 19,134 bp in length) and ECP.82 (contig 30; 53,048 bp in length). These two contigs had 97.2% identity in alignments of 11,523 in length. The genetic organization of the regions downstream of two *mcr-1*.*1* contigs shared the similar genes including genes encoding haemolysin expression modulating protein, DNA topoisomerase III, PcfJ domain-hypothetical protein and shufflon-specific DNA recombinase. When compared to plasmid pHNSHP45 (Accession number: KP347127), 99% nucleotide homology was found, covering the region of the published plasmid from 14,445–33,021 bp for ECP. 81 and 34,130–64,015 bp for ECP.82.

The IncI2 plasmid harboring *mcr-1*.*1* in ECP. 81 (64,006 bp in length) and ECP.82 (64,023 bp in length) were reconstructed based on the WGS data by comparison to plasmid pHNSHP45. The *mcr-1*.*1*-IncI2 plasmids had a typical plasmid backbone containing *repA* for plasmid replication, *tra*L for plasmid maintenance and transfer. Only an IS*Apl1*-*mcr-1*.*1* cassette identical to that of pHNSHP45 was found in ECP.81, whereas *mcr-1*.*1* of ECP.82 was flanked by IS*Apl1* and IS*91* transposase at upstream and downstream regions, respectively (IS*Apl1*-*mcr-1*.*1*-orf-IS*91*).

**Fig 1:**
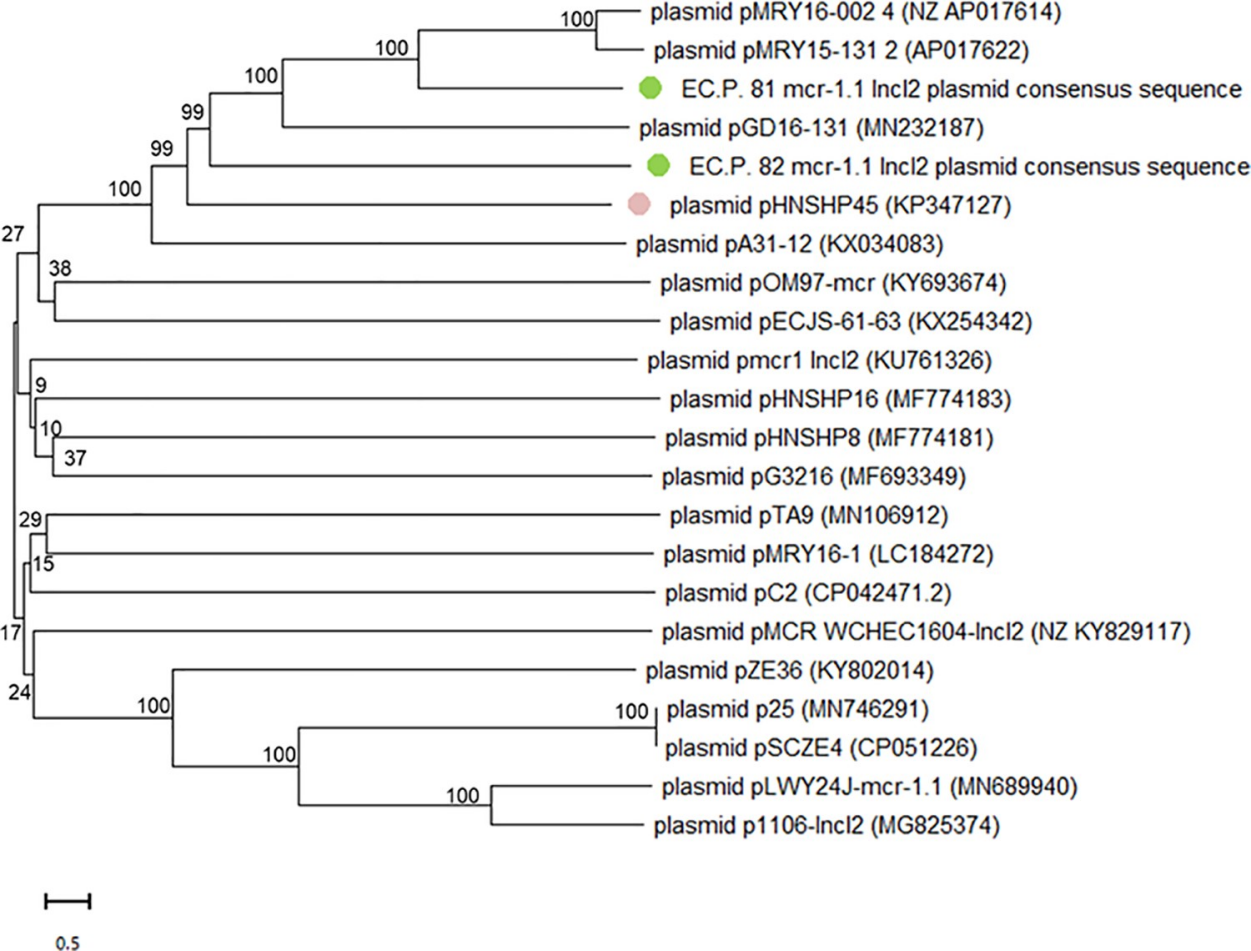
Phylogenetic analysis of the *mcr-1*.*1*-carrying IncI2 plasmids. Phylogenetic analysis of the *mcr-1*.*1*-carrying IncI2 plasmids in ECP.81 and ECP.82 from sick pigs (green dot) and other *mcr-1*-carrying IncI2 plasmids from *E*. *coli* deposited in the GenBank database. Sequences were aligned using MUSCLE and phylogenetic interferences were obtained using the neighbor-joining method within the MEGA 10 software. Numbers at the nodes are percentages of bootstrap values obtained by repeating the analysis for 1000 times to generate a majority consensus tree.

**Fig 2:**
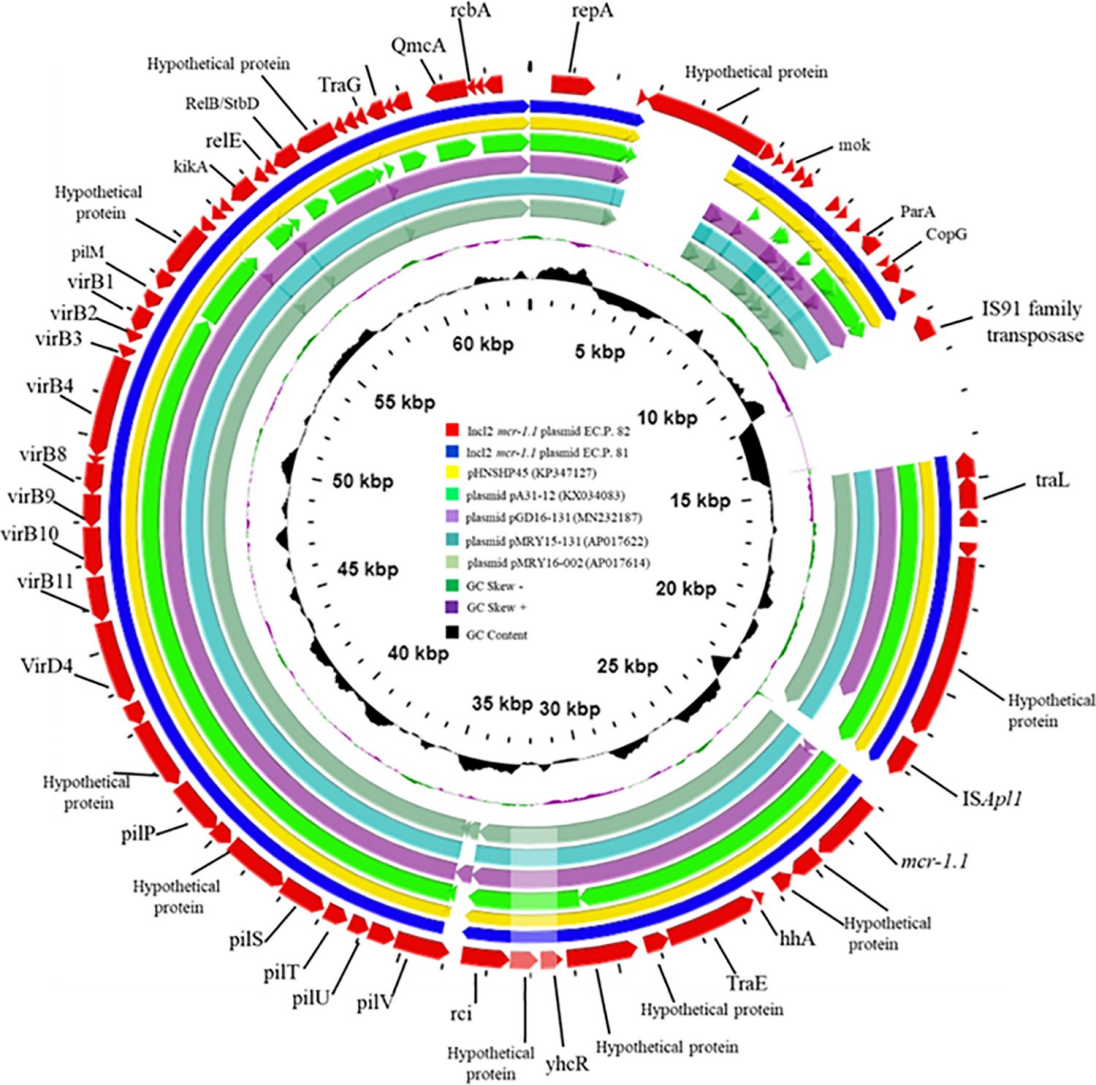
Circular comparison between *mcr-1*.*1*-carrying IncI2 plasmids from ECP.81 and ECP.82 to five IncI2 type plasmids carrying *mcr-1* with the highest similarity obtained from NCBI database. Circular comparison between *mcr-1*.*1*-carrying IncI2 plasmids from ECP.81 and ECP.82 to five IncI2 type plasmids carrying *mcr-1* with the highest similarity obtained from NCBI database (Accession No. KP347127, KX034083, MN232187, AP017622 and AP017614) generated by the Comparative Genomic tool (CG view) freely available at https://CGViewServer.ca. The outer-red circle denotes annotation of *mcr-1*.*1* plasmids from the present study. The sequencing alignment indicates the high degree similarity of the *mcr-1*.*1* harboring IncI2 plasmids from ECP.81 and ECP.82 to the *mcr-1*-harboring plasmid, pHNSHP45, isolated in China. The insertions element, IS*Apl1* is conserved in all *mcr-1*-containing plasmids. Gaps indicate regions that were missing in the respective plasmid compared to the reference plasmid.

Based on in silico phylogenetic analysis, phylogenetic of *mcr-1*.*1*-harboring IncI2 plasmids revealed 8 distinct clades (Fig 1), of which each clade consisted of DNA sequences from different origins. Both plasmid from ECP.81 and ECP.82 were found on the same clonal linages and clonally related to pHNSHP45. The *mcr-1*.*1*-harboring IncI2 plasmids identified in ECP. 81 and ECP. 82 were also similar to the *mcr-1* harboring IncI2 plasmids isolated from a cattle in Japan (pMRY16-002, Accession No. AP017622), a wild boar in China (pMRY15-131, Accession No. AP017614) and chicken in China (pGD16-131, Accession No. MN232187) (Fig 2).

The *mcr-2*.*1* gene was identified in E.431, of which only short assembled contig of 2,142 bp was generated. This *mcr-2*.*1* cassette contained a hypothetical protein at downstream and its entire length exhibited 90% identity to the 26687 to 28611 bp region of the published sequence of plasmid pKP37-BE in *E*. *coli* strain KP37 (Accession No. LT598652). No plasmid replicon and other AMR genes were detected on the same contig.

**Fig 3:**
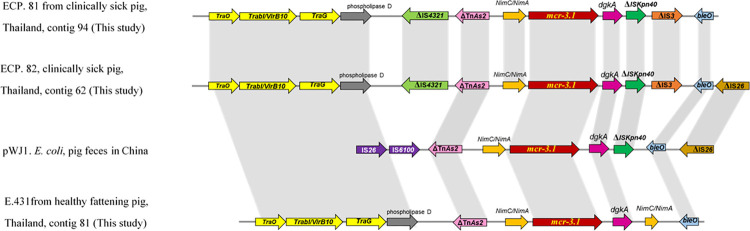
Comparative schematic representation of the flanking regions of the *mcr-3* gene in pWJ1 (Accession no. KY924928) and *mcr-3* carrying contigs in this study. The arrows indicate the positions and directions of the genes. The gray shade indicates homology in the corresponding genetic environment on each contig. AMR genes are indicated in red for *mcr-3*.*1* and blue for *bleO*, bleomycin resistance gene. The conjugally-transferred proteins are indicated in yellow. The green, pink, orange and brown arrows represent transposon-associated genes (ΔIS*4321*, ΔTn*As2*, ΔIS*Kpn40 and Δ*IS*26*).

All three porcine *E*. *coli* isolates in this study harbored *mcr-3*.*1*. The size of assembled contigs ranged from 8,412 to 11,172 bp. The WGS data showed that *mcr-3*.1 carrying plasmid in all the isolates had a backbone similar to *mcr-3*.*1*-carrying plasmid pWJ1 (Accession No. KY924928) (Fig 3). Five insertion sequences (IS) flanking the *mcr-3*.*1* cassette were identified. The upstream IS were IS*4321* (ECP.81 and ECP.82) and ΔTn*As2* (E.431, ECP.81 and ECP.82) and those at downstream were IS*Kpn40* and IS*3* family transposase (ECP.81 and ECP.82) and IS*26* (ECP.82). A core segment ΔTn*As2*- *NimC/NimA*-*mcr-3*.*1*-dgkA and conjugation transfer genes (i.e. *trb* and *traO*, and *traG*) were presented in all three *mcr-3*.*1*-carrying plasmid. The additional insertion sequence, IS*4321*, was immediately upstream of the ΔTn*As2*- *NimC/NimA*-*mcr-3*.*1*-*dgkA* segment in *mcr-3* carrying contigs of ECP.81 and ECP.82. None of the contigs contained other AMR genes and plasmid replicon type sequence identified in the PlasmidFinder database.

**Fig 4:**
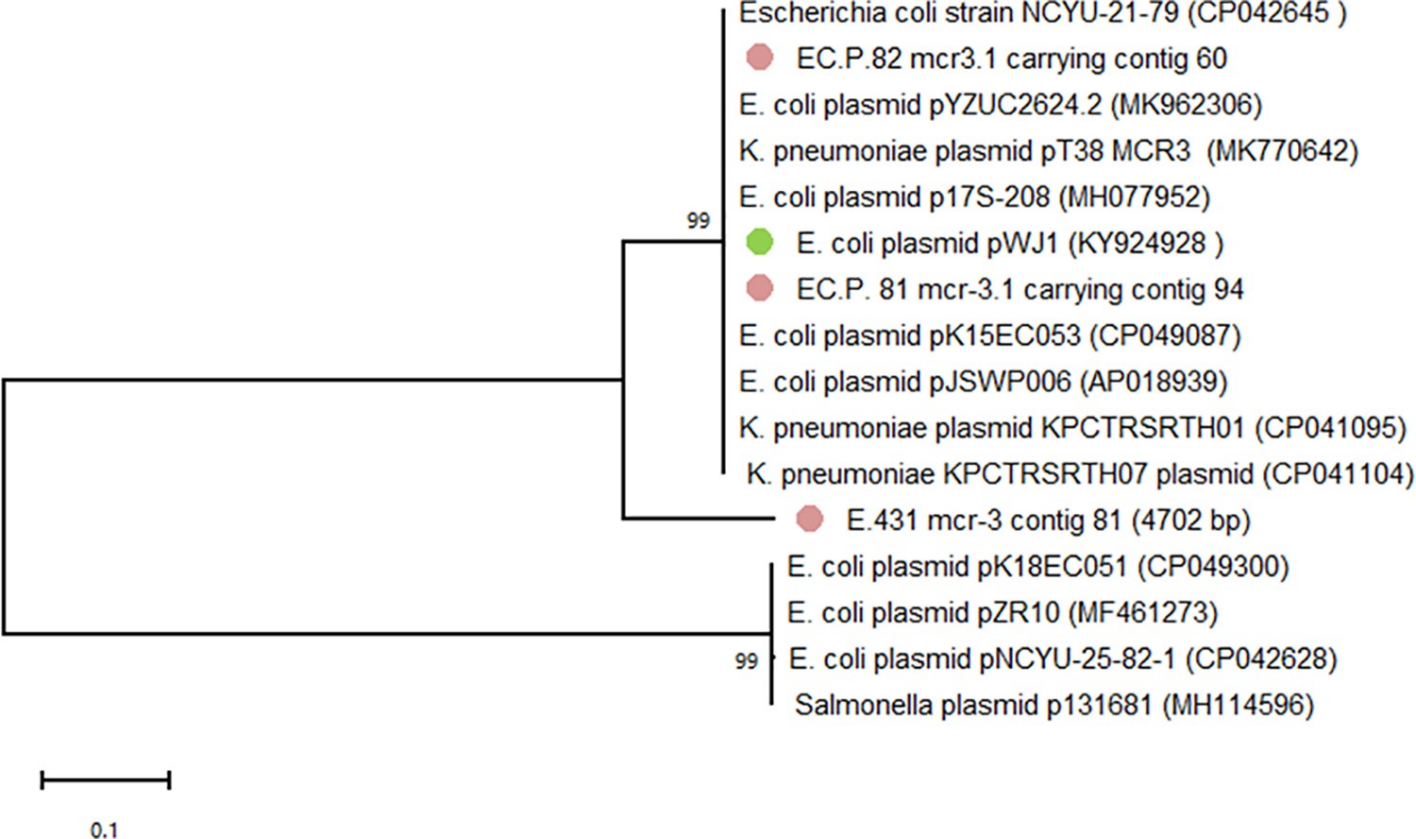
Phylogenetic tree of core genome sequences in E.431, ECP.81 and ECP.82 and other *mcr-3* plasmids deposited in the GenBank database. Sequences were aligned using MUSCLE and phylogenetic interferences were obtained using the maximum likelihood method within the MEGA 10 software. Numbers at the nodes are percentages of bootstrap values obtained by repeating the analysis of 1000 times to generate a majority of consensus tree.

Phylogenetic tree of the core genome sequences was analyzed in all three *mcr-3*.*1*-carrying isolates and 12 *mcr-3*.*1* harboring-plasmids deposited in the GenBank database (Fig 4). The members in this phylogenetic tree can be grouped into three clades. The *mcr*.*3*.*1* carrying contigs in ECP. 81 and EC.P.82 had a core segment that were similar to pWJ1 (Accession No. KY924928) isolated from in *E*. *coli* from pigs in China and in *Klebsiella pneumoniae* from pigs in Nakhon Pathom, Thailand (Accession No. CO041095 and CP041104). However, the *mcr-3*.*1* carrying contig from fattening pig (E.431) was different from those in sick pigs and reference plasmids.

## Discussion

The emergence and spread of colistin resistance mediated by plasmid-borne *mcr* genes has occurred globally, of which the majority was associated with bacterial pathogens including *E*. *coli*., *Moraxella* spp., *Klebsiella* spp., *Salmonella* spp., *Enterobacter* spp. *and Citrobacter* spp [44]. The *mcr*-carrying plasmids frequently co-harbor other resistance determinants e.g. *bla*_VIM-1_ [17] and *bla*_CTX-M1_ [45]. These raise a particular challenge for treatment of MDR Gram-negative bacterial infection due to limited availability of effective antibiotics. *E*. *coli*, particularly from pig, has been a major species among the *mcr*-carrying bacterial strains [10, 11, 46]. In the present study, we performed WGS analysis for molecular characterization of three ESBL producing *E*. *coli* additionally carrying *mcr* originated from pigs.

The *E*. *coli* isolates from healthy fattening pig, E.431, carrying *mcr-2/mcr-3* belonged to serotype O142:H31 and had sequence type, ST29. The other two isolates originated from clinically sick pigs, ECP.81 and ECP.82, with coexistence of *mcr-*1 and *mcr-3* appeared to be serotype O9:H9 with sequence type ST10. This supports previous studies demonstrating that the ST10 *E*. *coli* served as an important reservoir of *mcr-1* [46, 47]. The clonal group ST10 of *mcr*-carrying *E*. *coli* was reported previously in the clinical isolates from humans [48] and food-producing animals (i.e. poultry and swine) in Thailand [49] and other Asian countries [50, 51] as well as some European countries [16, 52]. It was shown that the ST10 strains harboring *mcr* commonly carried additional-multiple AMR genes e.g. *bla*_CTX-M1_ and *bla*_SHV-12_ [16], in agreement with the isolates in this study.

E.431, ECP.81 and ECP.82 were MDR, which exhibited resistance to different antimicrobial classes e.g. colistin, ß-lactam, aminoglycosides, fluoroquinolone, tetracycline and sulfa-trimethoprim. The AMR phenotype in all these isolates was consistent with AMR genotype revealed by WGS e.g. resistance to colistin (i.e. *mcr-1*.*1*, *mcr-2*.*1* and *mcr-3*), ß-lactams (i.e. *bla*_CTX-M14_, *bla*_CTX-M55_ and *bla*_TEM-1B_), aminoglycosides (i.e. *aadA1* and *aadA2*), fluoroquinolones (i.e. *oqxA*, *oqxB* and *qnrS13*), phenicols (i.e. *catA2* and *cmlA1*), tetracycline (i.e. *tetA* and *tetM*) and sulfamethoxazole (i.e. *sul2* and *sul3*). These results agree with previous studies demonstrating that colistin resistant bacteria commonly exhibited MDR phenotype and frequently co-harbored multiple AMR genes [44, 53].

The conjugation experiments and plasmid analysis confirmed that *mcr-1*.*1* of ECP.81 and ECP. 82 was located on IncI2 plasmid, in agreement with a previous study [10]. The *mcr-1* gene and its variants have been identified to be associated with four major plasmid incompatibility groups i.e. IncX4, IncI2, IncHI2 and ColE10-like [46, 47]. In Thailand, a previous study in clinical CRE isolates carrying *mcr-1* revealed that IncX4 was predominant replicon type, followed by IncI2 [54]. Taken together, these results suggest the circulation of IncI2 plasmid harboring *mcr-1* among bacterial species of animal and human origins. It was previously shown that IncI2 plasmid can migrate between different bacterial species and *E*. *coli* serves as a potential carrier of this plasmid replicon [55].

IS*Apl1* has been the main driver of mobilized colistin resistance gene, *mcr-1*, via horizontal gene transfer [56]. Four genetic contexts surrounding *mcr-1* cassette were identified including the composite transposon Tn6330 (IS*Apl1*–*mcr-1*–orf–*ISApl1*) and a single-IS*Apl1* located upstream (IS*Apl1*–*mcr-1*–orf); a single-IS*Apl1* (*mcr-1*–orf–IS*Apl1*) located downstream and a structure lacking both copies of IS*Apl1* (*mcr-1*–orf) [56]. In present study, the two *mcr-1*.*1* carrying IncI2 plasmids from ECP.81 and ECP. 82 had the structure of IS*Apl1*–*mcr-1*–orf. The results from the conjugation experiment and plasmid analysis in donor (ECP. 82) and transconjugant (ECP. 82T) confirmed that ISApl1 is associated with the mobilization of *mcr-1*. This transconjugant, ECP. 82T, carried *mcr-1*.*1* with translocation of IS*Apl1* to its upstream or IS*Apl1*–*mcr-1*–orf as observed in its donor strain. A recent study hypothesized that *mcr-1* translocation could be mediated through a circular intermediate that mediates the insertion of the *mcr-1* gene cassette into other bacterial plasmids or genome [19].

The *mcr-2* gene was first described on IncX4 plasmid in *E*. *coli* isolated from calves and piglets in Belgium [26]. However, the prevalence of *mcr-2* was low and limited to *E*. *coli*, *Moraxella* and *K*. *pneumoniae* from pigs, claves, bird, and chicken in some countries e.g. China [57], Belgium [26], USA and Great Britain [58]. Up to date, six *mcr-2* variants (Accession no. LT598652, MF176239, NG065452, MT757845, MT757842 and MT757844) were deposited on to GenBank database but only one plasmid sequence, pBK37-BE (Accession no. LT598652), was characterized. In our study, the *mcr-2*.*1* gene in E.431 showed 100% identity to pBK37-BE, with IS*1595* located upstream. However, analysis of genetic contexts of the genomic location *mcr-2* was limited due to the limited size of the contig and non-transferability in the conjugation experiment.

The *mcr-3* gene was first identified on pWJ1 that is a 261 kb IncHI2-type plasmid in *E*. *coli* from pigs in China [11]. Up to date, the gene was identified on IncP1, IncFII and IncI1 plasmids in many bacteria species e.g. *E*. *coli*, *K*. *pneumoniae*, *Salmonella*, *Enterobacter* spp. and *Aeromonas* spp. from pig, chicken, cattle, aquatic environment and human in several countires i.e. China [59], Denmark [60] and Spain [61]. In this study, only *mcr-3*.*1* in ECP. 81 was located on conjugative plasmid. Previous studies described that the dissemination of *mcr-3* was involved in mobile elements that can be horizontally transferred such as ΔTn*As2*, IS*Kpn40*, IS*26 and* IS*15DI* [20]. The mobile elements ΔTnAs2 and *Δ*IS*Kpn40* were originated from *Aeromonas* spp and may play an important role in the spread of *mcr-3* between *Aeromonas* spp. and Enterobacteriaceae [11]. In this study, five mobile elements were identified in the flanking regions of *mcr-3* contigs including ΔIS*4321*, ΔTn*As2*, ΔIS*Kpn40*, IS*26* and IS*3*. ECP.81 and ECP.82 contained a core segment of IS*4321*-ΔTn*pA*-*NimC/NimA*-*mcr-3*-*dgkA*-IS*Kpn40*-IS*3*-Δ*bleO*, while E. 431 carried ΔTn*pA*-*nimC/nimA*-*mcr-3*-*dgkA*-*nimC/nimA*-Δ*bleO*. The core segment ΔTn*pA*-*nimC/nimA*-*mcr-3*-*dgkA* in all three isolates was similar to pWJ1. However, ECP.81 and ECP.82 had IS*4321* instead of IS6100. This IS*4321* originated from *K*. *aerogenes* and was previously identified in *mcr-3* carrying plasmids, pZR78 (Accession no. MF455226) and pZR12 (Accession no. MF455227) [20].

ESBL genes are frequently located on conjugative plasmids and has been previously shown to be associated with several plasmid incompatibility groups e.g. IncF, IncI, IncH and IncA/C plasmids [62]. IncF is a commonly described plasmid type identified in humans and animals and *E*. *coli* is considered a major reservoir of this plasmid [62]. A previous study in Korea demonstrated the dissemination of *bla*_CTX-M14_ gene driven by IncF plasmid [63]. A study in China reported that *bla*_CTX-M55_ in *E*. *coli* from pets and food animals were linked to IncI2 plasmid [64]. Both IncHI1 and IncHI2 plasmids are frequently associated with resistance to multidrugs e.g. sulphonamides, aminoglycosides, tetracyclines and streptomycin in additions to cephalosporins. The *bla*_CTX-M2_ and *bla*_TEM-1_ genes were previously reported to be on a IncHI2 plasmid [65]. In this study, many plasmid replicon types were detected in the three *E*. *coli* strains, of which the most frequent plasmid replicons were IncFIB, IncFII and IncHI2. The *bla*_CTX-M14_ gene was detected in all *E*. *coli* isolates. The *bla*_CTX-M55_ gene was found only in ECP.81 and *bla*_TEM-1B_ was identified in both isolates from ill pigs. Four mobile elements (i.e. Tn3 transposase, Tn*903*, IS*1* and IS26) were identified in flanking regions of ESBL genes. This supports that, in addition to conjugative plasmid, the dissemination of ESBL genes may occur through transposons.

Moreover, the co-transfer of *mcr-1*.*1* and *bla*_TEM-1B_ was observed in ECP. 82 and the co-transfer of *mcr-3*.*1* and *bla*_CTX-M55_ was observed in ECP. 81. Using data from WGS analysis of the donor and plasmid analysis of transconjugants, plasmid reconstruction did not show the presence of other AMR genes. This implies that the genes encoding colistin resistance and ESBL production (i.e. *mcr-1*.*1*/ *bla*_TEM-1B_ and *mcr-3*.1/ *bla*_CTX-M55_) were not colocalized on the same plasmid with other AMR genes. These observations are in agreement with a previous study in China demonstrating the co-transfer of *mcr-1*.*1*, *mcr-3*.*5*, *bla*_NDM-5_ and *rmtB* could occur even though they were not located on the same plasmid [66]. The co-transfer of many clinically important AMR genes at the same time is a big challenge for clinical treatment and disease controlling in both human and veterinary medicine. Such co-transfer of multiple resistance genes could provide the fitness advantage to the host strains [67]. Therefore, using colistin alone may not be the only reason of *mcr* dissemination. This is additionally supported by the use of ampicillin as selective pressure in conjugative experiment in this study, where colistin resistance gene(s) was co-selected.

In conclusion, the pandemic spread of *mcr* genes is attributed to several factors e.g. MDR profile in colistin-resistant strains, host fitness adaptation, and co-selection by other antimicrobials. Co-transfer of *mcr* and ESBL genes has generated a challenging for clinical treatment and a serious concern to global health. Judicious use of antibiotics in livestock and human medicine should be encouraged. Characterization of the coexistence ESBL and *mcr* genes in three *E*. *coli* isolates by using WGS approach provided large database for rapid analysis of genetic context of resistance determinants and supported better understanding on the dissemination of these resistance markers among bacteria from different sources. However, a short-read sequencing platform was used in this study and deemed insufficient to elucidate complete sequence and genetic organization of plasmid. Therefore, long read sequencing approach (e.g. Pacific Biosciences and Oxford Nanopore Technologies) producing long reads with higher-quality genome assemblies and thus improving de novo assembly is suggested for further study.

## Supporting information

S1 Table Primers used in this study. (DOCX)

S2 Table Information of referent sequences used in this study. (DOCX)
