# Supplementary material for: Whole genome sequencing and characteristics of Escherichia coli with co-existence of ESBL and mcr genes from pigs
Source: PLoS One. 2021 Nov 16;16(11):e0260011. doi: 10.1371/journal.pone.0260011 (PMC8594834; doi:10.1371/journal.pone.0260011)
Supplement: S1 Table — (DOCX) [file pone.0260011.s001.docx]

| PCR and primer names | | Sequence (5’ – 3’) | Amplicon size (bp) | Reference |
| --- | --- | --- | --- | --- |
| *Mcr* genes | |  |  |  |
| MCR1-IF | | CGGTCAGTCCGTTTGTTC | 309 | [1] |
| MCR1-IR | | CTTGGTCGGTCTGTA |  |  |
| MCR2-IF | | TGTTGCTTGTGCCGATTGGA | 619 | [2] |
| MCR2-IR | | AGATGGTATTGTTGGTTGCTG |  |  |
| MCR3-IF | | TTGGCACTGTATTTTGCATTT | 542 | [3] |
| MCR3-IR | | TTAACGAAATTGGCTGGAACA |  |  |
| MCR4-IF | | ATTGGGATAGTCGCCTTTTT | 487 | [4] |
| MCR4-IR | | TTACAGCCAGAATCATTATCA |  |  |
| β-lactamase and ESBL genes | |  |  |  |
|  | *bla*_CTX-M__FW | CGATGTGCAGTACCAGTAA | 585 | [5] |
|  | *bla*_CTX-M__RW | AGTGACCAGAATCAGCGG |  |  |
|  | *bla*_PSE-M__FW | GCTCGTATAGGTGTTTCCGTTT | 575 | [6] |
|  | *bla*_PSE-M__RW | CGATCCGCAATGTTCCATCC |  |  |
|  | *bla*_TEM__FW | GCGGAACCCCTATTT | 964 | [7] |
|  | *bla*_TEM__RW | TCTAAAGTATATATGAGTAAACTTGGTCT |  |  |
|  | *bla*_SHV__FW | TTCGCCTGTGTATTATCTCCCTG | 854 | [8] |
|  | *bla*_SHV__RW | TTAGCGTTGCCAGTGYTG |  |  |
|  | *bla*_CMY-1__FW | GTGGTGGATGCCAGCATCC | 854 |  |
|  | *bla*_CMY-1__RW | GGTCGAGCCGGTCTTGTTGAA |  |  |
|  | *bla*_CMY-2__FW | GCACTTAGCCACCTATACGGCAG | 856 |  |
|  | *bla*_CMY-2__RW | GCTTTTCAAGAATGCGCCAGG |  |  |
|  | Multiplex CTX-M group 1 and group 2 |  |  | [9] |
|  | MultiCTXMGp1_FW | TTAGGAARTGTGCCGCTGYA^a^ | 688 |  |
|  | MultiCTXMGp1_RW | CGATATCGTTGGTGGTRCCAT^a^ |  |  |
|  | MultiCTXMGp2_FW | CGTTAACGGCACGATGAC | 404 |  |
|  | MultiCTXMGp2_RW | CGATATCGTTGGTGGTRCCAT^a^ |  |  |
|  | CTX-M group 8/25_FW | AACRCRCAGACGCTCTAC^a^ | 326 |  |
|  | CTX-M group 8/25_RW | TCGAGCCGGAASGTGTYAT^a^ |  |  |
|  | CTX-M group 9_FW | GTGACAAAGAGAGTGCAACGG | 850 | [10] |
|  | CTX-M group 9_RW | ATGATTCTCGCCGCTGAAGCC |  |  |
|  | CTX-M15_SFW | CACACGTGGAATTTAGGGACT | 876 | [11] |
|  | CTX-M15_SRW | GCCGTCTAAGGCGATAAACA |  |  |

**Supporting information table 1.** Primers used in this study.

^a^ Y = T or C; R = A or G; S = G or C; D=A, G, or T

1. Liu YY, Wang Y, Walsh TR, Yi LX, Zhang R, Spencer J, et al. Emergence of plasmid-mediated colistin resistance mechanism MCR-1 in animals and human beings in China: a microbiological and molecular biological study. The Lancet Infectious diseases. 2016;16(2):161-8. doi: 10.1016/S1473-3099(15)00424-7. PubMed PMID: 26603172.

2. Xavier BB, Lammens C, Ruhal R, Kumar-Singh S, Butaye P, Goossens H, et al. Identification of a novel plasmid-mediated colistin-resistance gene, mcr-2, in Escherichia coli, Belgium, June 2016. Euro surveillance : bulletin Europeen sur les maladies transmissibles = European communicable disease bulletin. 2016;21(27). doi: 10.2807/1560-7917.ES.2016.21.27.30280. PubMed PMID: 27416987.

3. Yin W, Li H, Shen Y, Liu Z, Wang S, Shen Z, et al. Novel Plasmid-Mediated Colistin Resistance Gene mcr-3 in Escherichia coli. mBio. 2017;8(3). doi: 10.1128/mBio.00543-17. PubMed PMID: 28655818; PubMed Central PMCID: PMC5487729.

4. Carattoli A, Villa L, Feudi C, Curcio L, Orsini S, Luppi A, et al. Novel plasmid-mediated colistin resistance mcr-4 gene in Salmonella and Escherichia coli, Italy 2013, Spain and Belgium, 2015 to 2016. Euro surveillance : bulletin Europeen sur les maladies transmissibles = European communicable disease bulletin. 2017;22(31). doi: 10.2807/1560-7917.ES.2017.22.31.30589. PubMed PMID: 28797329; PubMed Central PMCID: PMC5553062.

5. Batchelor M, Hopkins K, Threlfall EJ, Clifton-Hadley FA, Stallwood AD, Davies RH, et al. bla(CTX-M) genes in clinical *Salmonella* isolates recovered from humans in England and Wales from 1992 to 2003. Antimicrobial agents and chemotherapy. 2005;49(4):1319-22. doi: 10.1128/AAC.49.4.1319-1322.2005. PubMed PMID: 15793104; PubMed Central PMCID: PMC1068621.

6. Li R, Lai J, Wang Y, Liu S, Li Y, Liu K, et al. Prevalence and characterization of *Salmonella* species isolated from pigs, ducks and chickens in Sichuan Province, China. International journal of food microbiology. 2013;163(1):14-8. doi: 10.1016/j.ijfoodmicro.2013.01.020. PubMed PMID: 23474653.

7. Olesen I, Hasman H, Møller Aarestrup F. Prevalence of β-lactamases among ampicillin-resistant *Escherichia coli* and *Salmonella* isolated from food animals in Denmark. Microb Drug Resist. 2004;10(4):334-40.

8. Hasman H, Mevius D, Veldman K, Olesen I, Aarestrup FM. beta-Lactamases among extended-spectrum beta-lactamase (ESBL)-resistant *Salmonella* from poultry, poultry products and human patients in The Netherlands. J Antimicrob Chemother. 2005;56(1):115-21. Epub 2005/06/09. doi: 10.1093/jac/dki190. PubMed PMID: 15941775.

9. Dallenne C, Da Costa A, Decre D, Favier C, Arlet G. Development of a set of multiplex PCR assays for the detection of genes encoding important beta-lactamases in Enterobacteriaceae. The Journal of antimicrobial chemotherapy. 2010;65(3):490-5. doi: 10.1093/jac/dkp498. PubMed PMID: 20071363.

10. Sabate M, Tarrago R, Navarro F, Miro E, Verges C, Barbe J, et al. Cloning and sequence of the gene encoding a novel cefotaxime-hydrolyzing beta-lactamase (CTX-M-9) from Escherichia coli in Spain. Antimicrobial agents and chemotherapy. 2000;44(7):1970-3. PubMed PMID: 10858363; PubMed Central PMCID: PMC89994.

11. Muzaheed, Doi Y, Adams-Haduch JM, Endimiani A, Sidjabat HE, Gaddad SM, et al. High prevalence of CTX-M-15-producing *Klebsiella pneumoniae* among inpatients and outpatients with urinary tract infection in Southern India. J Antimicrob Chemother. 2008;61(6):1393-4. Epub 2008/03/22. doi: 10.1093/jac/dkn109. PubMed PMID: 18356153; PubMed Central PMCID: PMCPMC2736628.

**References**
