# Supplementary material for: Whole genome sequencing and characteristics of Escherichia coli with co-existence of ESBL and mcr genes from pigs
Source: PLoS One. 2021 Nov 16;16(11):e0260011. doi: 10.1371/journal.pone.0260011 (PMC8594834; doi:10.1371/journal.pone.0260011)
Supplement: S2 Table — (DOCX) [file pone.0260011.s002.docx]

**Supporting information table 2.** Information of referent sequences used in this study.

| Sample ID | *mcr* gene | Species | Collection Date | Country of isolates | Nucleotide Accession(s) | Source |
| --- | --- | --- | --- | --- | --- | --- |
| Plasmid pMRY16-002 | *mcr-1* | *E. coli* strain: 20Ec-P-124 | 2008 | Japan | AP017614 | Wild boar |
| Plasmid pMRY15-131_2 | *mcr-1* | *E. coli* strain: MRY15-131 | 2013 | Japan | AP017622 | Cattle |
| Plasmid pGD16-131 | *mcr-1* | *E. coi* pGD16-131 | 2019 | China | MN232187 | Chicken |
| Plasmid pHNSHP45 | *mcr-1* | *E. coli* strain SHP45 | 2015 | China | KP347127 | Swine |
| Plasmid pOM97-mcr | *mcr-1* | *E. coli* train OM97 | 2016 | United Arab Emirates | KY693674 | Human |
| Plasmid pECJS-61-63 | *mcr-1* | *E. coli* strain JS-61 | 2015 | China | KX254342 | Swine |
| Plasmid pmcr1 IncI2 | *mcr-1* | *E. coli* strain SZ02 | 2016 | China | KU761326 | Human |
| Plasmid pHNSHP16 | *mcr-1* | *E .coli* strain SHP16 | 2017 | China | MF774183 | Swine |
| Plasmid pHNSHP8 | *mcr-1* | *E. coli* strain SHP8 | 2017 | China | MF774181 | Swine |
| Plasmid pG3216 | *mcr-1* | *E. coli* strain 3216 | 2016 | Argentina | MF693349 | Human |
| Plasmid pTA9 | *mcr-1* | *E. coli* strain TA9 | 2017 | China | MN106912 | Duck meat |
| Plasmid pMRY16-1 | *mcr-1* | *E. coli* pMRY16 | 2016 | Japan | LC184272 | ND |
| Plasmid pC2 | *mcr-1* | *E. coli* strain A50 | 2017 | Algeria | CP042471 | Chicken |
| Plasmid pMCR WCHEC1604-IncI2 | *mcr-1* | *E. coli* strain WCHEC1604 | 2017 | China | KY829117 | Human |
| Plasmid pZE36 | *mcr-1* | *E. coli* strain ZE36 | 2015 | China | KY802014 | Human |
| Plasmid p25 | *mcr-1* | *E. coli* strain GN2982 | 2019 | Ecuador | MN746291 | Dog |
| Plasmid pSCZE4 | *mcr-1* | *E. coli* strain SCZE5 | 2019 | China | CP051226 | Swine |
| Plasmid pLWY24J-mcr-1.1 | *mcr-1* | *E. coli* strain LWY24J | 2016 | China | MN689940 | Chicken |
| Plasmid p1106-IncI2 | *mcr-1* | *E. coli* strain 1106 | 2018 | China | MG825374 | Chicken |
| Plasmid pKP37-BE | *mcr-2* | *E. coli* strain KP37 | 2016 | Belgium | LT598652 | Swine |
| NCYU-21-79 | *mcr-3* | *E. coli* strain NCYU-21-79 | 2017 | Taiwan | CP042645 | Swine |
| Plasmid pYZUC2624.2 | *mcr-3* | *E.coli* strain YZUC2624 | 2017 | China | MK962306 | Swine |
| Plasmid pT38_MCR-3 | *mcr-3* | *K. pneumoniae* strain T38 | 2019 | China | MK770642 | Human |
| Plasmid p17S-208 | *mcr-3* | *E. coli* strain EC17S-208 | 2017 | South Korea | MH077952 | Swine |
| Plasmid pK15EC053 | *mcr-3* | *E. coli* strain pV01-15-R02-025-053 | 2015 | South Korea | CP049087 | Swine |
| Plasmid pJSWP006_1 | *mcr-3* | *E. coli* strain *JSWP006* | 2015 | Japan | AP018939 | Wastewater |
| Plasmid KPCTRSRTH01 | *mcr-3* | *K. pneumoniae* strain KPCTRSRTH01 | 2016 | Thailand | CP041095 | Swine |
| Plasmid KPCTRSRTH07 | *mcr-3* | *K. pneumoniae* strain KPCTRSRTH07 | 2016 | Thailand | CP041104 | Swine |
| Plasmid pK18EC051 | *mcr-3* | *E. coli* strain pV01-18-E02-025-051 | 2018 | South Korea | CP049300 | Swine |
| plasmid pZR10 mcr-3 | *mcr-3* | *E. coli* strain WZR10 | 2017 | China | MF461273 | Swine |
| Plasmid pNCYU-25-82-1_MCR3 | *mcr-3* | *E. coli* strain NCYU-25-82 | 2017 | Taiwan | CP042628 | Swine |
| Plasmid p131681 | *mcr-3* | *Salmonella enterica* strain 13-1681 | 2018 | Canada | MH114596 | Human |
